# Stroke and transient ischemic attack recurrence after PFO closure in patients with cryptogenic embolism

**DOI:** 10.1007/s12471-026-02042-w

**Published:** 2026-04-08

**Authors:** Matthijs W. Kanon, Elke S. Hoendermis, Erik A. Badings, Jan van Wijngaarden

**Affiliations:** 1 Department of Cardiology, Deventer Hospital, Deventer, The Netherlands; 2 Department of Cardiology, University Medical Centre Groningen, Groningen, The Netherlands

**Keywords:** Ischemic Stroke, Patent Foramen Ovale

## Abstract

**Background:**

Patent foramen ovale (PFO) closure is recommended in selected patients with a cryptogenic stroke and a PFO, as multiple randomized controlled trials (RCTs) have shown superiority of PFO closure over medical therapy in reducing recurrent stroke risk. However, follow-up data outside clinical trials remains scarce. The aim of this study is to evaluate the stroke/transient ischemic attack (TIA) recurrence after PFO closure.

**Methods:**

Data from 242 patients who underwent PFO closure at the University Medical Centre Groningen (UMCG) between February 2016 and June 2023, because of a cryptogenic stroke, TIA, or peripheral embolism, were collected.

**Results:**

During a median follow-up of 3.1 years (IQR: 1.9–4.3), a total of 5 recurrent strokes (0.70 per 100 person-years) and 11 recurrent TIAs (1.54 per 100 person-years) were documented. In patients with a recurrent neurological event, hypertension was more prevalent (30.8% vs. 9.6%, *p* = 0.040). Atrial fibrillation (AF) or atrial flutter was seen in 5.7% of the patients. Other adverse events were rare.

**Conclusion:**

In this study, the rate of recurrent stroke/TIA after PFO closure was low, but higher than reported in previous studies. These elevated values are primarily due to the higher risk of recurrent TIA, as the recurrent stroke risk in our cohort is comparable to that reported in RCTs. Hypertension was significantly associated with the recurrence of a stroke/TIA.

## What’s new?

- This study supports the safety and efficacy of PFO closure for secondary stroke prevention.
- While PFO closure is effective for secondary stroke prevention, the usefulness of PFO closure to prevent recurrent TIA is debatable.
- Monitoring and controlling of individual cardiovascular risk factors seems essential for secondary stroke prevention.

## Introduction

Stroke is one of the major causes of disability-adjusted life years worldwide and the second most common cause of death in Europe [1]. In approximately 10% of all strokes, no other evident cause is identified except a patent foramen ovale (PFO) [2]. Guidelines recommend transcatheter PFO closure in selected patients with a cryptogenic stroke and a PFO, as multiple randomized controlled trials (RCTs) have shown superiority of PFO closure over antiplatelet therapy in reducing recurrent stroke risk [2–13]. An individual participant data meta-analysis, consisting of all major RCTs, showed recurrent stroke rates of 1.09% a year in the medical therapy group and 0.47% a year in the PFO closure group, a 59% relative reduction [14]. However, the absolute reduction was moderate at 0.62%, and PFO closure has its risks and adverse effects [15]. Furthermore, it is uncertain whether this superiority is generalizable to the daily clinical practice, as RCTs have strict eligibility criteria and have excluded patients who are often included in daily clinical practice, such as patients above 60 years old. Limited data exist on clinical outcomes after PFO closure outside clinical trials [16]. Hence, the objective of this study is to analyse the stroke/TIA recurrence and adverse events after PFO closure in daily clinical practice in the Netherlands.

## Methods

### Study population and eligibility

All patients who received transcatheter PFO closure in the UMCG between February 2016 and June 2023, because of a cryptogenic stroke, TIA, or peripheral embolism, were included. Patients undergoing PFO closure for other indications were excluded. Data were collected from medical records between February 2016 and September 2024. Most patients were referred from general hospitals to the UMCG. These patients underwent screening in the general hospital: laboratory tests for anticoagulation disorders, rhythm monitoring for detecting AF for a minimum duration of 72 h, brain MRI and/or brain CT, and transcarotid Doppler or CT angiography of the carotid arteries.  PFO was diagnosed using transthoracic echocardiography (TTE) with an agitated saline contrast test, performed both with and without the Valsalva maneuver.

Patients were considered eligible for PFO closure if they had a RoPE score of ≥ 6, or < 6 with high-risk PFO features, like a large shunt or the presence of an atrial septum aneurysm (ASA), and/or a clinical condition highly suggestive of paradoxical embolism across a PFO, like a Valsalva situation at the moment of stroke. Patients with AF, a coagulation disorder, or another potential cause of stroke were considered ineligible. The final decision regarding eligibility was made in an interdisciplinary meeting with at least one cardiologist and one neurologist. The procedures were performed under general anaesthesia or conscious sedation and with transoesophageal echocardiography (TEE) and fluoroscopic guidance. All implanted devices were Occlutech Figulla Flex II devices, and device size was determined by the physician performing the procedure. TTE was performed the day after the procedure to control the device’s position.

### Follow-up

Follow-up at the UMCG was performed 2 to 4 weeks post-PFO closure with a TTE. At 3–6 months, the follow-up was performed either in the UMCG or in the referring hospital. After 3–6 months, further follow-up was in the judgment of the referring cardiologist. Follow-up data were requested from the general hospitals. In the case of recurrent neurological events, neurologist’s correspondence and neuroimaging data were enquired. In every patient whose last follow-up visit was before 2023, a systematic phone call was conducted for information about recurrent neurological events, bleeding, arrhythmias, and antithrombotic medication. If the patient reported an event, neurologist’s correspondence was requested from the corresponding hospital.

### Statistical analysis

All patient data were registered in Epic Hyperspace or Chipsoft HiX. A database was created, and statistical analysis was performed using IBM SPSS Statistics, version 29.0.1.0. Categorical variables were reported as *n* (%). Depending on the variable distribution, continuous variables were reported as mean ± SD or median (25th to 75th interquartile range). Group comparisons were analysed using the Student’s *t*-test or Mann-Whitney U test for continuous variables and chi-square test or Fisher’s exact test for categorical variables. Kaplan-Meier curves were used to analyse time-to-event data. Results were considered significant at *p* < 0.05.

### Ethical review

The Medical Ethical Review Committee confirmed that the Medical Research Involving Human Subjects Act (WMO) was not applicable, and this study was approved by the Central Ethical Review Committee of the UMCG.

## Results

### Baseline and procedural characteristics

**Fig. 1:**
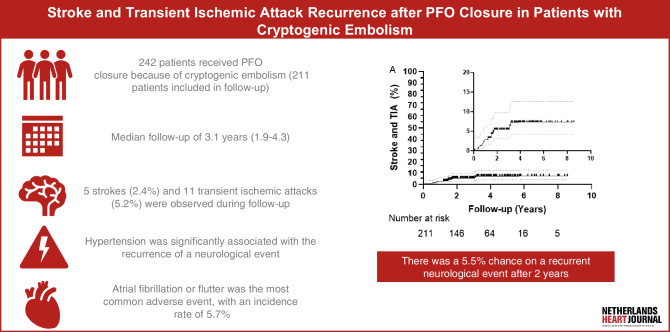
Infographic illustrating the recurrent neurological events and adverse events

A total of 242 patients received PFO closure for a cryptogenic embolism (Fig. 1). The mean age of the patients was 45 (± 11) years and 133 patients (55%) were male. 17 patients were over 60 years old. The incidence of cardiovascular risk factors was 18.2% for smoking, 9.5% for hypertension, and 2.1% for diabetes mellitus. The mean RoPE score was 7.2 (± 1.3). The indication for PFO closure was a stroke in 172 (71.1%) patients, TIA in 67 (27.7%) patients, and a peripheral embolism in 3 (1.2%) patients (Tab. 1).

**Table 1:** Baseline and procedural characteristics

*N* = 242	
Age, yrs	45 ± 11
*Sex*	
– Male	133 (55.0)
– Female	109 (45.0)
Body mass index, kg/m^2^	25.4 (23.0–27.8)
Smoking	44 (18.2)
Hypertension	23 (9.5)
Diabetes Mellitus	5 (2.1)
*Closure indication*	
– Stroke	172 (71.1)
– TIA	67 (27.7)
– Peripheral embolism	3 (1.2)
Multiple neurological events	37 (15.1)
RoPE score	7.2 ± 1.3
Successful implantation	241 (99.6)
Device size, mm *	
– 16/18	21 (8.7)
– 23/25	168 (69.7)
– 27/30	42 (17.4)
– 31/35	8 (3.3)
– Unknown	2 (0.8)
Residual shunt at discharge*	6 (2.5)
*Periprocedural complications*	
– AF	0 (0.0)
– Device embolisation	0 (0.0)
– Device thrombosis	1 (0.4)
– Cardiac tamponade	0 (0.0)
– Oesophageal hematoma	1 (0.4)
– Anaphylactic shock	1 (0.4)
– Bleeding at access site	6 (2.4)
*Antithrombotic treatment at discharge*	
– Aspirin + Clopidogrel	192 (79.7)
– Aspirin	2 (0.8)
– Clopidogrel	2 (0.8)
– VKA	18 (7.5)
– NOAC	26 (10.8)
– NOAC + Aspirin	1 (0.4)

Values are *n* (%), mean ± standard deviation or median (25th–75th percentile)

*RoPE* Risk of Paradoxical Embolism, *TIA* transient ischemic attack

* *N* = 241

An Occlutech device was successfully implanted in 241 patients (99.6%), and 9 complications were documented. During one intervention, a device thrombosis was seen in the left atrium at TEE despite adequate antithrombotic therapy. The thrombus resolved the next day with anticoagulation. One patient had an oesophageal hematoma after intervention, although hemodynamically stable and with full recovery. Another patient had an anaphylactic shock after administration of cefazolin. There were no cases of periprocedural death, AF, device embolisation, or cardiac tamponade.

### Follow-up

**Fig. 2:**
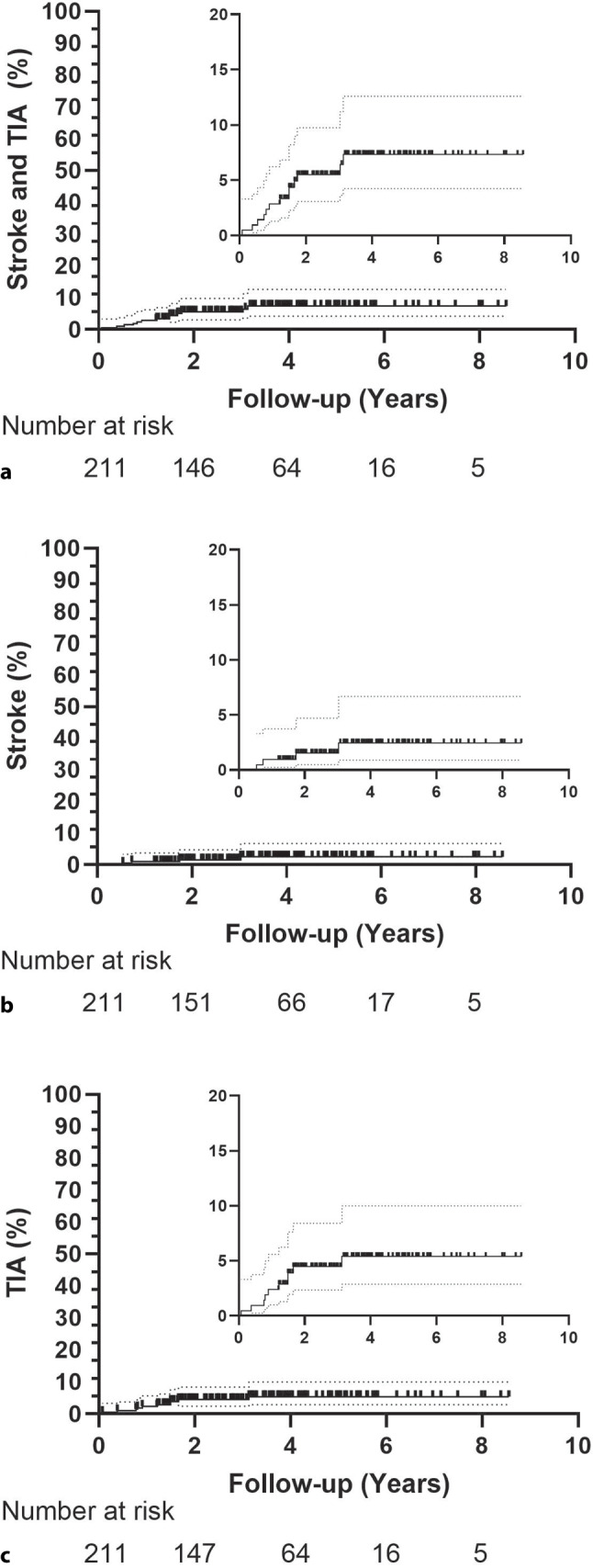
Kaplan-Meier curves showing the **a** combined risk of stroke and TIA; **b** risk of stroke; and **c** risk of TIA during follow-up. *N* = 211, *TIA* transient ischemic attack

30 patients were lost to follow-up and excluded from the analysis, as their last follow-up data were recorded prior to 2023, and no additional data were available. Median follow-up was 3.1 years (1.9–4.3). During follow-up, 13 patients had a recurrent neurological event, with a total of 16 events. 5 strokes (0.70 per 100 person-years) and 11 TIAs (1.54 per 100 person-years) were documented (Tab. 2). Three patients had more than one recurrent event. Kaplan-Meier curves are shown in Fig. 2. The estimated combined risk of stroke and TIA was 2.8%, 5.5%, and 7.4% at 1, 2, and 5 years, respectively, with stroke risk of 0.9%, 1.5%, and 2.5%, and TIA risk of 2.4%, 4.5%, and 6.4% over the same time periods.

**Table 2:** Clinical outcomes at follow-up

*N* = 211	
Follow-up, yrs	3.1 (1.9–4.3)
Death	1 (0.5)
Number of patients with stroke and/or TIA	13 (6.2)
Total number of recurrent neurological events	16 (7.6)
Stroke	5 (2.4)
TIA	11 (5.2)
AF/atrial flutter	12 (5.7)
Venous thromboembolism	3 (1.4)
Peripheral embolism	1 (0.5)
Myocardial infarction	1 (0.5)
Bleeding	6 (2.8)
*Other adverse event*	
– Residual shunt requiring 2nd device closure	1 (0.5)
– Residual shunt treated with device removal and surgical closure	1 (0.5)
– Device thrombosis	1 (0.5)

Values are *n* (%) or median (25th–75th percentile)

*TIA* transient ischemic attack, *AF* atrial fibrillation

### Adverse events

One patient died during follow-up due to an unknown cause. AF or atrial flutter was observed in 12 patients (5.7%). In 8 patients, AF or atrial flutter developed within 45 days of the procedure, and only one case was persistent. One patient had a peripheral embolism in the right foot, the symptoms resolved without intervention. One patient had a device endocarditis with thrombus on the device on both atrial sides 2 years after intervention, requiring surgical removal of the device and thrombus and closure of the PFO. Examination of the device revealed that there was incomplete endothelialisation of the device. One patient had a residual shunt, which needed surgical closure. However, 5 years after closure, the patient had another TIA. Bleedings were reported in 6 patients (2.8%), of which 3 were gynaecologic and 3 were severe epistaxis. No intracranial bleedings were observed.

### Neurological recurrence

Patient characteristics stratified by recurrence of a neurological event are presented in Tab. 3. Patients with a recurrent neurological event had a higher prevalence of hypertension (30.8% vs. 9.6%, *p* = 0.040), and there was a trend towards a lower RoPE score (6.5 vs. 7.2), this was not statistically significant (*p* = 0.057). No further significant differences were observed.

**Table 3:** Patient characteristics stratified by the recurrence of a neurological event

	*No recurrent stroke/TIA* *(N* *=* *198)*	*Recurrent stroke/TIA* *(N* *=* *13)*	*p Value*
Age, yrs	45 ± 11	48 ± 11	0.284
Age > 60 yrs	12 (6.1)	2 (15.4)	0.209
Male	111 (56.1)	9 (69.2)	0.402
Body mass index, kg/m^2^	25.7 (23.4–27.9)	25.9 (24.0–30.4)	0.525
Smoking	37 (18.7)	2 (15.4)	1.000
Hypertension	19 (9.6)	4 (30.8)	*0.040*
Diabetes Mellitus	3 (1.5)	1 (7.7)	0.226
Multiple neurological events	29 (14.6)	4 (30.8)	0.126
*Closure indication*			
– Stroke	142 (71.7)	8 (61.5)	0.528
– TIA	53 (26.8)	5 (38.5)	0.522
– Peripheral embolism	3 (1.5)	0 (0.0)	1.000
RoPE score	7.2 ± 1.3	6.5 ± 1.4	0.057

Values are *n* (%), mean ± standard deviation or median (25th–75th percentile)

*TIA* transient ischemic attack, *RoPE* Risk of Paradoxical Embolism

## Discussion

In our study, we observed a recurrent stroke rate of 0.70 per 100 person-years and TIA rate of 1.54 per 100 person-years after PFO closure. In patients with a recurrent event, there was a higher prevalence of hypertension. Furthermore, there was a trend towards a lower RoPE score. However, this was not significant.

To our knowledge, only a few studies investigated the clinical outcomes after PFO closure outside RCTs [16, 18–22]. These RCTs have shown low recurrent stroke rates after PFO closure, with a pooled incidence rate of 0.47 per 100-person years [13, 14]. Our study shows slightly higher recurrence rates than the RCTs. A potential cause can be the fact that RCTs have strict eligibility criteria, whereas observational studies investigate routine clinical practice. To illustrate, we included patients with TIA as an index event and patients above 60 years of age, if other criteria suggestive of a PFO-associated neurological event were present. Those patients were excluded from most RCTs.

The combined recurrent stroke/TIA rate (2.24 per 100-person years) in our study is also higher compared to other studies. With a mean RoPE score of 7.2, a 6% chance of a recurrent neurological event in 2 years was expected with medical therapy, as estimated by the publication of Kent et al. [23]. Our Kaplan-Meier estimate shows a 5.5% chance of recurrent neurological events after 2 years in our cohort. When analysed separately, the estimated 2‑year risk was 1.5% for stroke and 4.5% for TIA. The 2‑year stroke risk in our cohort aligns with findings from a meta-analysis, including all major RCTs [13]. This suggests that the higher risk of recurrent neurological events in our cohort is mainly attributable to the higher risk of TIA. The meta-analysis showed no significant reduction in TIA risk after PFO closure compared with antiplatelet therapy [13]. Due to its transient nature, lack of biomarkers, and the overlap of clinical presentation with other conditions such as migraine, accurately diagnosing a TIA can be challenging [24]. With this in mind, it is reasonable to consider that the recurrent TIAs observed in our study may, in fact, represent another diagnosis, despite thorough neurological evaluation by a neurologist.

In our study, hypertension was significantly associated with the recurrence of a neurological event. This finding aligns with the study of Sørensen et al. [22] As also stated by Goessinger et al. [20], age accounts for 50% of the total RoPE score, whereas other individual risk factors only account for 10%. While the RoPE score provides valuable risk stratification, it may underemphasize the importance of individual cardiovascular risk factors, such as hypertension, and overemphasize the influence of age. Furthermore, the RoPE score does not include high-risk PFO features, like a large shunt or the presence of an ASA, which studies have demonstrated to be associated with an increased risk of recurrent stroke [15, 25]. We suggest that greater emphasis should be placed on evaluating individual, circumstantial, and anatomical risk factors, rather than relying exclusively on the total RoPE score. The PASCAL score, which includes anatomical risk factors and the ROPE score, is therefore now widely used in decision-making [14]. Moreover, strict control and monitoring of cardiovascular risk factors seem essential for secondary stroke prevention after PFO closure.

For patients above 60 years old, no clear recommendations are given in guidelines, as most RCTs excluded elderly patients. In our study, the rate of recurrent stroke/TIA did not differ significantly between patients aged below and above 60 years. This aligns with previous studies [20, 26]. We suggest that PFO closure may be safe for carefully selected patients over 60 years old and without cardiovascular risk factors, especially when high-risk PFO features are present. However, future RCTs are needed to prove this hypothesis.

Our study shows great safety of PFO closure in terms of periprocedural complications. Long-term complications of PFO closure were mainly AF or atrial flutter, which was seen in 12 patients (5.7%). This number is comparable to other studies, although incidence numbers differ slightly between different devices (4.7–7.6%). [5, 27–29] 8 out of 12 patients developed AF or atrial flutter within 45 days after the procedure, of which only one was persistent. Although this is not classified as a periprocedural complication, it strongly suggests an association with the procedure. These findings support the idea that AF after PFO closure tends to be transient, and the long-term risk of AF appears to be low [30].

### Limitations

Our study has several limitations. First, a relatively large group of patients was lost to follow-up. Secondly, only major bleedings during follow-up are documented. Minor bleedings, therefore, could be missed, and the total amount of bleedings can be underestimated. Thirdly, due to the fact that primary outcomes and adverse events are infrequent, subgroup analyses are small. However, our study is one of the few studies providing real-world data on the recurrence of neurological events after PFO closure. Large multicenter studies are needed to enable more robust and well-defined subgroup analysis. Finally, analysis of the PASCAL score was not possible, as its regular documentation began only recently in our hospital. However, its criteria have already been used for decision-making for a long time.

## Conclusion

The recurrent stroke/TIA rate after PFO closure in our cohort was low, but higher than reported in previous studies. These elevated values are primarily due to the relatively higher risk of recurrent TIA, as the recurrent stroke risk in our cohort is comparable to that reported in RCTs. The rate of adverse events was low. These findings support the safety and efficacy of PFO closure for secondary stroke prevention. We suggest that, after PFO closure, more emphasis should be placed on the monitoring of individual cardiovascular risk factors such as hypertension, which was significantly associated with the recurrence of a recurrent neurological event. Large, multicenter studies should be conducted to create more insight into the risk of neurological recurrence after PFO closure outside RCTs.
